# Regulator of G Protein Signaling 20 Correlates with Long Intergenic Non-Coding RNA (lincRNAs) Harboring Oncogenic Potential and Is Markedly Upregulated in Hepatocellular Carcinoma

**DOI:** 10.3390/biology11081174

**Published:** 2022-08-04

**Authors:** Yulu Wang, Maria F. Setiawan, Hongde Liu, Tikam Chand Dakal, Hongjia Liu, Fangfang Ge, Oliver Rudan, Peng Chen, Chunxia Zhao, Maria A. Gonzalez-Carmona, Miroslaw T. Kornek, Christian P. Strassburg, Matthias Schmid, Jarek Maciaczyk, Amit Sharma, Ingo G. H. Schmidt-Wolf

**Affiliations:** 1 Department of Integrated Oncology, Center for Integrated Oncology (CIO), University Hospital Bonn, 53127 Bonn, Germany; 2 State Key Laboratory of Bioelectronics, School of Biological Science & Medical Engineering, Southeast University, Nanjing 210096, China; 3 Genome and Computational Biology Lab, Department of Biotechnology, Mohanlal Sukhadia University, Udaipur 313001, India; 4 School of Nursing, Nanchang University, Nanchang 330006, China; 5 Department of Internal Medicine I, University Hospital Bonn, 53127 Bonn, Germany; 6 Institute of Medical Biometry, Informatics and Epidemiology, University Hospital Bonn, 53127 Bonn, Germany; 7 Department of Neurosurgery, University Hospital Bonn, 53127 Bonn, Germany

**Keywords:** liver cancer, regulator of G protein signaling 20, the cancer genome atlas, long intergenic non-coding RNA, prognosis, biomarker

## Abstract

**Simple Summary:**

Clinical and molecular advances have improved knowledge and treatment prospects for cancer, yet hepatocellular carcinoma (HCC), the most common form of liver cancer, still ranks significantly higher in terms of the global cancer burden. Herein, we investigated the role of RGS20 as a potential prognostic marker in 28 different cancers with a particular focus on HCC.

**Abstract:**

Hepatocellular carcinoma (HCC) is at the forefront of the global cancer burden, and biomarkers for HCC are constantly being sought. Interestingly, RGS (Regulators of G protein signaling) proteins, which negatively regulate GPCR signaling, have been associated with various cancers, with some members of the RGS family being associated with liver cancer as well. Considering this, we investigated the role of RGS20 as a potential prognostic marker in 28 different cancer types with special emphasis on HCC. By using the Cancer Genome Atlas (TCGA) and Gene Expression Omnibus (GEO) data, our analysis revealed that (a) RGS20 was strongly upregulated in tumor tissue compared with adjacent normal tissue of HCC patients; (b) RGS20 was strongly associated with some important clinical parameters such as alpha-fetoprotein and tumor grade in the HCC patients; (c) besides HCC (*p* < 0.001), RGS20 was found to be an important factor for survival in four other cancers (clear renal cell carcinoma: *p* < 0.001, lung adenocarcinoma: *p* = 0.004, mesothelioma: *p* = 0.039, ovarian serous cystadenocarcinoma: *p* = 0.048); (d) RGS20 was found to be significantly associated with some tumor-related signaling pathways and long intergenic non-coding RNAs (lincRNAs: LINC00511, PVT1, MIR4435-2HG, BCYRN1, and MAPKAPK5-AS1) that exhibit oncogenic potential. Taken together, we showed that RGS20 correlates with a few HCC-associated lincRNAs harboring oncogenic potential and is markedly upregulated in HCC patients. Our analysis further supports the putative function of RGS proteins, particularly RGS20, in cancer.

## 1. Introduction

Liver cancer is the fourth leading cause of cancer-related deaths [1], and remains a global health concern among prevalent cancers, being hepatocellular carcinoma (HCC) the most common form of liver cancer. In the last few decades, the number of liver cancer cases has also increased, primarily due to hepatitis C infection and nonalcoholic fatty liver disease (NAFLD).

Indeed, clinical and molecular advances have improved the knowledge and treatment perspective, still, HCC ranks significantly higher in terms of global cancer burden. While many approaches have been applied to the treatment of HCC, cancer immunotherapy has been at the forefront of respective clinical trials and patient care [2,3]. There has been a constant search for identifying HCC-related biomarkers, however, most of them showed association with poor prognosis, either in early or advanced HCC [4]. Notably, two members of the ubiquitin C-terminal hydrolases (UCHs) family, BRCA1-associated protein-1 (BAP1) [5] and UCH-L3 [6] have been implicated in the survival rate of this particular cancer. More recently, authors established immuno-autophagy-related long non-coding RNA (IARlncRNA) signature with a prognostic ability in HCC [7]. Recently, the crucial role of G protein-coupled receptors (GPCRs) in tumorigenesis and HCC development has been discussed [8]. Interestingly, RGS (Regulators of G protein signaling) proteins, which negatively regulate GPCR signaling, have been implicated in various cancers including lung, prostate, breast, and ovarian cancers [9,10,11,12]. To date, 20 canonical RGS genes (RGS1-RGS20) have been reported and a few members of the RGS family (RGS3, RGS5, RGS17) have also been associated with liver cancer [13,14,15,16,17,18]. 

Considering this, herein, we focused our analysis on RGS20, which is solely associated with the occurrence and progression of several cancers, including breast cancer, bladder cancer, oral squamous cell carcinoma, and metastatic melanoma [19,20,21,22]. With special emphasis on HCC, we investigated the role of RGS20 as a potential prognostic marker. Furthermore, we also evaluated the survival probability of RGS20 in 28 different cancer types. In addition, we correlate its expression with putative HCC-related long intergenic non-coding RNAs (lincRNAs). To our knowledge, our study is the first to expand the clinical relevance and molecular significance of RGS20 in the cancer spectrum, especially HCC.

## 2. Materials and Methods

### 2.1. Gene Expression Data and Clinical Data

Gene expression data (workflow type: HTSeq—FPKM) was obtained from the TCGA-LICH dataset in the Cancer Genome Atlas Program (TCGA) database, which contains 374 tumor samples and 50 normal samples. The clinical data of HCC patients were downloaded from the GDC TCGA Liver Cancer (LIHC) dataset in the UCSC XENA database. The clinical data parameters included age, sex, Child-Pugh classification, alpha-fetoprotein (AFP), fibrosis, grade, and stage. Of note, 371 primary HCC samples were included in our analysis by excluding 3 recurrent samples. Among them, 365 samples contained survival data (survival time and survival status) and gene expression data, while 163 samples contained survival data, clinical characteristics and gene expression data. In addition, the gene expression data and clinical data of HCC in the GSE76427 dataset from the Gene Expression Omnibus (GEO) database were used for validation, which contains 115 tumor samples and 52 normal samples. Besides gene expression data, all 115 tumor samples also contained survival data. In addition, gene expression data (TPM) and cancer samples with overall survival data for 28 cancer types were obtained from TCGA database as well. These 28 cancer types include adrenocortical carcinoma (ACC, 73 samples), bladder urothelial carcinoma (BLCA, 400 samples), breast invasive carcinoma (BRCA, 1033 samples), cervical squamous cell carcinoma and endocervical adenocarcinoma (CESC, 292 samples), cholangiocarcinoma (CHOL, 33 samples), colon adenocarcinoma (COAD, 261 samples), lymphoid neoplasm diffuse large B-cell lymphoma (DLBC, 46 samples), esophageal carcinoma (ESCA, 182 samples), glioblastoma multiforme (GBM, 162 samples), head and neck squamous cell carcinoma (HNSC, 518 samples), kidney chromophobe (KICH, 60 samples), kidney renal clear cell carcinoma (KIRC, 509 samples), kidney renal papillary cell carcinoma (KIRP, 281 samples), acute myeloid leukemia (LAML, 102 samples), liver hepatocellular carcinoma (LIHC, 344 samples), lung adenocarcinoma (LUAD, 477 samples), lung squamous cell carcinoma (LUSC, 482 samples), mesothelioma (MESO, 79 samples), ovarian serous cystadenocarcinoma (OV, 418 samples), pancreatic adenocarcinoma (PAAD, 178 samples), pheochromocytoma and paraganglioma (PCPG, 179 samples), prostate adenocarcinoma (PRAD, 460 samples), stomach adenocarcinoma (STAD, 377 samples), testicular germ cell tumors (TGCT, 135 samples), thyroid carcinoma (THCA, 508, samples), thymoma (THYM, 118 samples), uterine corpus endometrial carcinoma (UCEC, 172 samples), uterine carcinosarcoma (UCS, 56 samples). The cutoff values for grouping patients into high and low RGS20 gene expression were based on the median value of RGS20 gene expression in each kind of cancer, respectively.

### 2.2. Gene Set Enrichment Analysis

Gene set enrichment analysis (GSEA) was used to determine a defined set of genes that exhibit statistical significance and consistent differences between the two biological states (e.g., phenotypes). In GSEA analysis, RGS20 expression was divided into low and high groups, and the cut-off value was considered as the median value of its expression. Gene set permutations were performed 1000 times for each analysis in the h.all.v7.4.symbols.gmt [Hallmarks] set. The expression level of RGS20 was set as a phenotype label. The enrichment of pathways in each phenotype was selected according to the *p* value < 0.05 and false discovery rate (FDR) < 0.25.

### 2.3. Prediction of RGS20 Interaction with lincRNAs

Both RGS20 gene expression (FPKM) and lincRNAs gene expression (FPKM) in LIHC were obtained from TCGA data and a total of 1193 lincRNAs were involved in this analysis. The averages of gene expression no more than 0 were excluded. Log2 was further applied to the gene expression data (FPKM) in order to obtain a suitable normalized distribution. Subsequently, gene expression (log2 (FPKM+1)) was used to investigate the correlation between RGS20 and lincRNAs using the Spearman correlation test. Statistical significance was determined using Spearman correlation coefficient |R| > 0.4 and *p* value < 0.05.

The prediction of physical and functional interaction between five lincRNAs and the RGS20 protein was performed in RNA-Protein Interaction Prediction (RPISeq, http://pridb.gdcb.iastate.edu/RPISeq/, accessed on 5 January 2022) using the protein sequence of RGS20 and RNA sequence of the lincRNAs. The output, i.e., prediction probability of possible interactions were obtained in terms of RF and SVM classifiers. Interaction probabilities range from 0 to 1, wherein the higher the probability is better. In general, prediction probabilities with scores of more than 0.5 are considered “positive,” i.e., expressing the likelihood of interaction between given lincRNA and protein. 

LincRNAs and RGS20 mRNA Interaction and Tissue-specific Expression Profile were investigated. The prediction of physical and functional interaction between five lincRNAs and the mRNA of RGS20 was performed using LncRRIsearch web server (http://rtools.cbrc.jp/LncRRIsearch/, accessed on 5 January 2022), which also gives tissue-specific expression level of lincRNAs and mRNA based on RNA-seq data from the Genotype-Tissue Expression (GTEx) Project (E-MTAB-2919). 

### 2.4. Statistical Analysis 

The statistical analyses were performed using R. The relationship between clinical characteristics and RGS20 was analyzed using Wilcoxon Rank Sum and logistic regression. The Kaplan–Meier method was used to demonstrate the association between RGS20 expression and overall survival (OS). Clinical variables and RGS20 were associated with survival using Cox regression. Multivariable Cox analysis was used to find independent factors based on the clinical characteristics and RGS20 gene. The cutoff value for RGS20 expression was determined by its median value. Spearman analysis was used to find lincRNAs related to RGS20. *p* values of less than 0.05 were considered statistically significant.

## 3. Results

### 3.1. RGS20 Gene Expression, Clinical Features Relevant and Survive Probability in HCC

**Figure 1:**
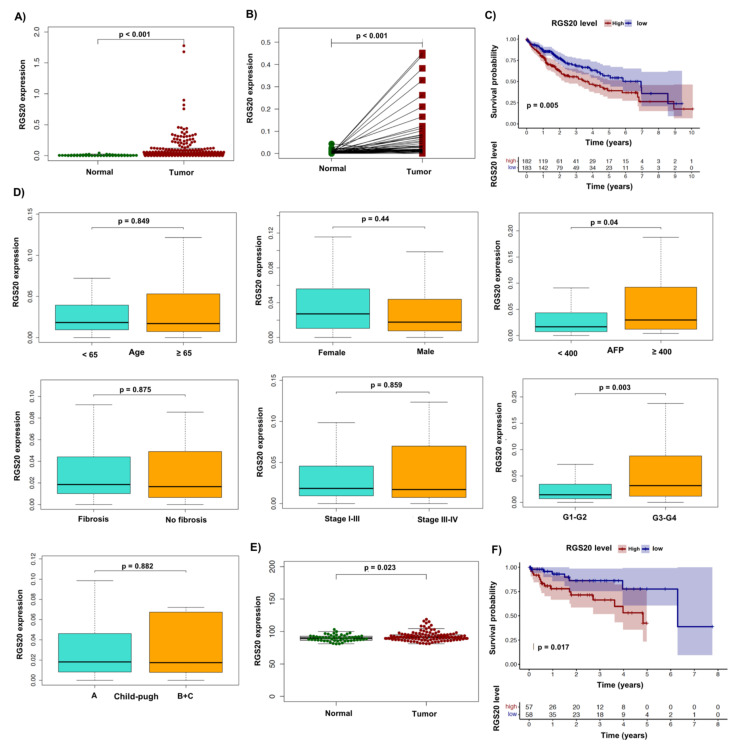
RGS20 analysis in HCC patients (TCGA and GEO analysis). (**A**) All samples, (**B**) and paired samples from TCGA data were analyzed using Wilcoxon Rank Sum test. (**C**) The impact of RGS20 expression level on overall survival time in HCC patients calculated using Kaplan–Meier method. (**D**) Relationship between RGS20 expression and clinical features using Wilcoxon Rank Sum test. (**E**) RGS20 gene expression between tumor and normal samples (Wilcoxon Rank Sum test), (**F**) and KM curve was used to assess the survival rate between high and low RGS20 expression group using GEO data.

We first sought to analyze the difference in RGS20 gene expression between normal and tumor tissue samples from the TCGA database. In the panel of 421 samples (tumor = 371, normal = 50) and paired samples (tumor = 50, normal = 50), we observed elevated expression of RGS20 in tumor samples compared to the controls (Figure 1A,B) using Wilcoxon Rank Sum test. To assess the survival pattern according to the RGS20 expression, we next divided the data into high and low expression groups (based on their median value) and analyzed them using Kaplan–Meier survival analysis (Figure 1C). The analysis showed that patients with higher RGS20 expression had a worse prognosis (*p* = 0.005), suggesting that RGS20 may be predictive of survival in liver cancer patients. Next, we correlate the patient-specific clinical parameters with the RGS20 expression. We specifically distinguished these clinical parameters in groups such as age (≥65 vs. <65), AFP (≥400 vs. <400), Child-pugh (B + C vs. A), fibrosis (no fibrosis vs. fibrosis), sex (male vs. female), grade (G3 + G4 vs. G1 + G2), stage (III + IV vs. I + II). Using Wilcoxon Rank Sum test, we found that RGS20 expression significantly correlated with AFP (*p* = 0.04) and grade (*p* = 0.003) (Figure 1D). Likewise, logistic regression analysis also confirmed this in the case of AFP (*p* = 0.043) and grade (*p* = 0.009) (Table 1). To verify the predictive function, we also used the independent dataset GSE76427 from the GEO database. The analysis clearly showed that the expression of RGS20 varied significantly between tumor and normal samples (*p* = 0.023) (Figure 1E). Importantly, the KM curve result also indicated that the group with higher RGS20 expression had a lower survival rate than the group with low expression (Figure 1F).

### 3.2. RGS20 Survive Probability Spectrum in 28 Cancers

**Figure 2:**
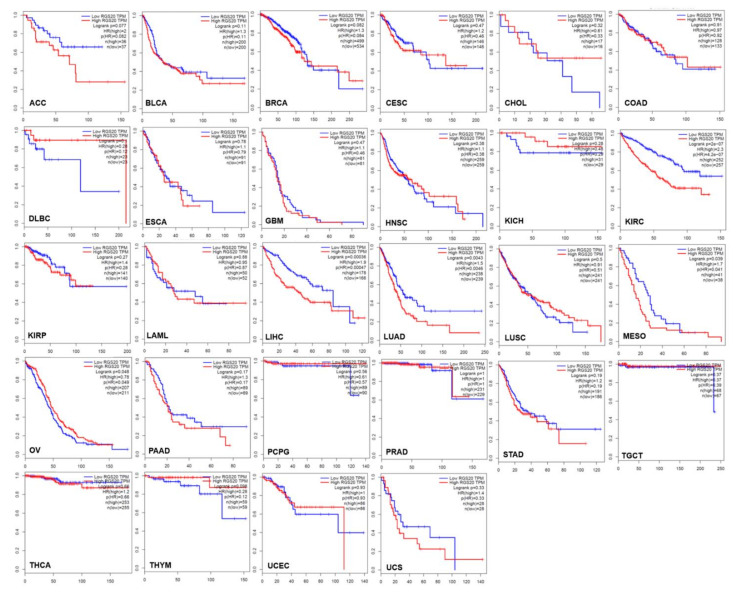
RGS20 survival probability in 28 cancers. KM curves show data from 28 cancers. Patients were classified into low and high expression groups based on the median value of RGS20 in each cancer.

To investigate the survival potential of RGS20 in other cancer types, we extend our analysis to 28 cancer types (Figure 2). Interestingly, only five cancers, namely KIRC (*p* < 0.001), LIHC (*p* < 0.001), LUAD (*p* = 0.004), MESO (*p* = 0.039), and OV (*p* = 0.048), showed a difference in survival between the high and low RGS20 expression groups. Of these, KIRC, LIHC, LUAD, and MESO cancers demonstrated poorer survival in the high RGS20 expression group, while OV cancers were observed to have poorer survival in the low RGS20 expression group.

### 3.3. Identification of Independent Factors and GSEA Enrichment Results 

**Figure 3:**
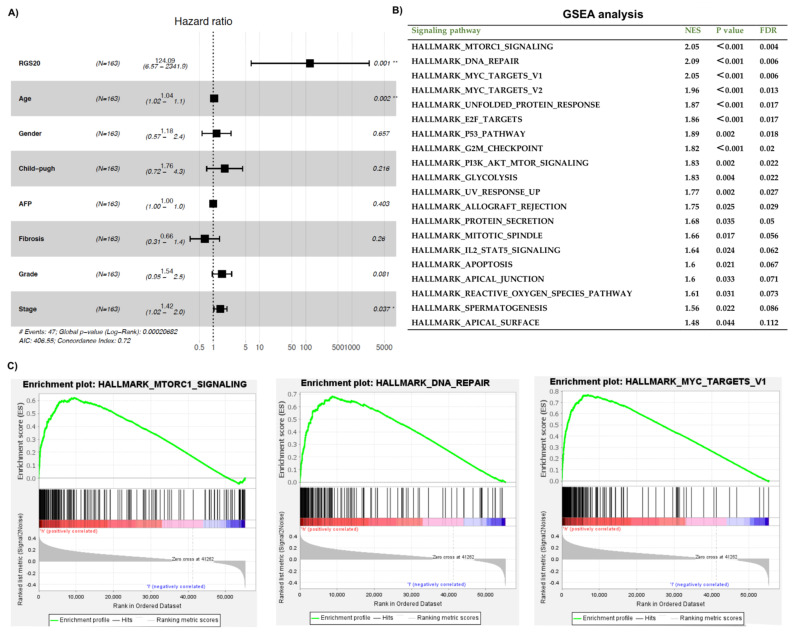
Multivariable Cox regression and GSEA enrichment analysis. (**A**) Multivariable Cox survival model including RGS20 and clinical features. The hazard ratio values are represented by squares. The horizontal bars depict the 95% CI of the hazard ratio estimation. * *p* < 0.05, ** *p* < 0.01. (**B**) GSEA enrichment results. (**C**) Top 3 Enrichment plots from GSEA (NES > 2). The green curves depict the enrichment score curve obtained from GSEA software. NES: normalized enrichment score; *p*-value: normalized *p*-value; FDR: false discovery rate.

In the context of overall survival (OS), univariate analysis also revealed that age (*p* = 0.022), stage (*p* = 0.017), and RGS20 (*p* < 0.001) were related to OS. (Table 2). In addition, multivariable Cox regression confirmed that age, stage, and RGS20 were independent factors associated with survival (Figure 3A). In the above part, each clinical feature was classified into two subgroups. However, age (continuous value), AFP (continuous value), RGS20 gene expression (continuous value), Child-pugh (A, B and C), fibrosis (no fibrosis and fibrosis), sex (female and male), grade (G1, G2, G3 and G4) and stage (I, II, III and IV) were applied in this part.

To determine whether RGS20 is involved in established biological pathways, we performed gene set enrichment analysis (GSEA) using the TCGA dataset. We first divided the RGS20 data into high and low cohorts and investigated them using hallmark gene sets. GSEA revealed that 20 gene signatures were enriched in patients with high RGS20 expression (FDR < 0.25, normalized *p*-value < 0.05) (Figure 3B). Some tumor-related pathways were included such as mTORC1, MYC TARGETS V1, MYC TARGETS V1, DNA REPAIR, P53, G2M CHECKPOINT, PI3K/AKT/MTOR, IL2/STAT5, and APOPTOSIS. Of which, DNA REPAIR, mTORC1, MYC TARGETS V1 signaling were the top three enriched terms with NES values > 2. These three enrichment plots are shown in Figure 3C.

### 3.4. Prediction of RGS20 Interaction with lincRNAs

**Figure 4:**
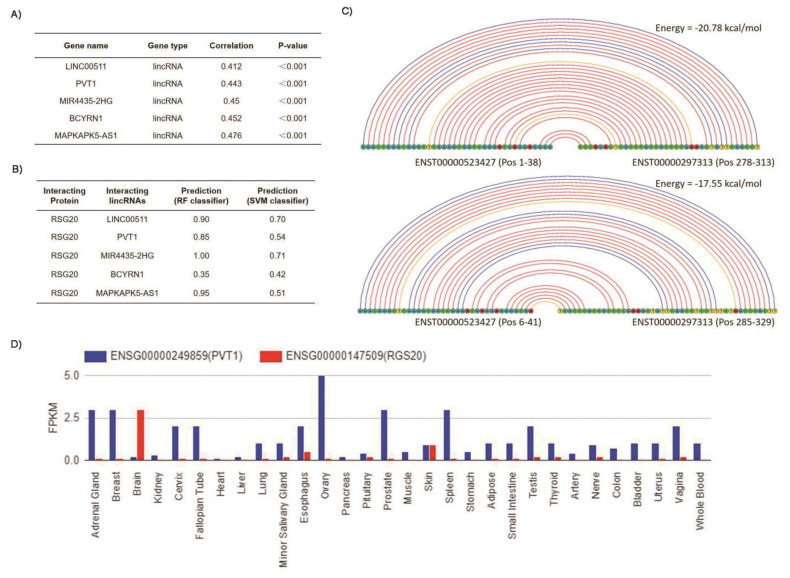
Prediction of RGS20 interaction with lincRNAs and gene expression in human tissues. (**A**) Correlation of RGS20 with five lincRNAs. (**B**) Interaction of five lincRNAs with RGS20 protein. (**C**) Two physical interactions of lincRNA PVT1 and mRNA of RGS20. (**D**) Gene expression level of the lincRNA PVT1 and the RGS20 mRNA in different tissues of humans.

Given that the non-coding genome has been suggested to contribute to the regulation of RGS protein in oral squamous cell carcinoma [22], cervical cancer [23], ovarian cancer [24] and lung cancer [25]. We therefore investigated the possible links of RGS20 to lincRNAs implicated in HCC. Using TCGA data, we performed Spearman correlation analysis (|R| > 0.4 and *p* < 0.05) and identified five lincRNAs, including LINC00511, PVT1, MIR4435-2HG, BCYRN1, and MAPKAPK5-AS1 in this category (Figure 4A).

Further investigations for the possible interaction of RGS20 with the obtained five lincRNAs were performed using two kinds of web tools. First, the prediction of physical and functional interaction between five lincRNAs and the RGS20 protein was performed using the RPISeq webtool of the Iowa State University [26]. The prediction probabilities in terms of RF and SVM classifiers of individual interaction of lincRNAs with the RGS20 protein have been shown in Figure 4B. The prediction probabilities indicate that all five obtained lincRNAs are likely to interact with RGS20 protein, except the lincRNA BCYRN1 (prediction probability less than the threshold value). Then, we subjected the five lincRNAs and RGS20 to the LncRRIsearch web server to predict possible physical and functional interaction. However, the information on lincRNA BCYRN1 was not available in this web tool which means it cannot predict the interaction of lincRNA BCYRN1 with mRNA of RGS20. For the remaining four lincRNAs, we found that only lincRNA PVT1 (Transcript ID: ENST00000523427) interacts with the mRNA of RGS20 (Transcript ID: ENST00000297313). The genomic locus of PVT1 (transcript length 938 nt) is chr8(+) 127,794,575–127,890,952 based on the UCSC Genome Browser (https://genome.ucsc.edu/, accessed on 5 January 2022). The genomic locus of RGS20 (transcript length 2104 nt) is chr8(+) 53,851,808–53,959,303. The total genomic distance is 73,835,271 bp. Figure 4C shows that two physical interactions of LincRNA PVT1 and RGS20 mRNA are possible with energy −20.78 and −17.55 kcal/mol, respectively. Of note, the energy threshold was set as −16 kcal/mol. In addition, we also checked the RNA expression level of the lincRNA PVT1 and the RGS20 mRNA. We generated the RNA-seq expression profile using the database E-MTAB-2919, which encompasses the expression profile of RNAs from different tissues in humans. The RNA-seq expression profile shows that the expression of PVT1 is very high in the ovary (FPKM ~5.0) and high in some other tissues such as the adrenal gland, breast, prostate and spleen (FPKM ~3.0) (Figure 4D). Compared to this the expression levels of RGS20 mRNA is almost negligible (FPKM~0.1–0.2) in these five tissues (Figure 4D). However, in brain tissue the expression of RGS20 mRNA is high (FPKM ~3.0) and the expression of lincRNA PVT1 is very low (FPKM ~0.2–0.3) (Figure 4D). 

## 4. Discussion

There has been a plethora of evidence to suggest that each cancer is unique and that there is considerable overlap in altered mutational pathways across the cancer genome [27,28,29]. Liver cancer is no different from other cancers in this respect, exhibiting shared molecular mechanisms. Interestingly, alterations in this particular gene have also been observed in numerous other cancers [30]. Due to clinical and molecular heterogeneity, stratification of patients remains a difficult task, especially in HCC, the predominant form of liver cancer. Of interest, several abnormally regulated signaling pathways [31], and the frequently mutated drivers [32] have been associated with HCC; however, their transformation as molecular therapy is still pending. Therefore, there is an urgent need to find more effective diagnostic and prognostic markers.

Since there have been recent discussions about the crucial role of G protein-coupled receptors (GPCRs) in tumorigenesis and the development of HCC [8]. Moreover, the possible involvement of RGS (Regulators of G protein signaling) proteins that negatively regulate GPCR signaling in various cancers. Herein, we investigated the potential role of RGS20 in liver cancer. As aforementioned, a few members of the RGS family (RGS3, RGS5, RGS17) have also been associated with liver cancer, however, the putative role of RGS20 as a prognostic indicator in HCC has not yet been investigated. We found that RGS20 was strongly upregulated in tumor tissue compared with adjacent normal tissue of HCC patients. In addition, RGS20 was strongly associated with some important clinical parameters such as AFP and grade in HCC patients. Of interest, RGS20 was found to be an important factor in the survival of HCC patients. Specifically, in TCGA data high RGS20 expression group was associated with a worse survival rate compared to the low RGS20 expression group. Using Cox regression analysis to examine independent HCC survival-related factors, some features including RGS20, age and tumor stage were confirmed. We also validated the prognostic potential of RGS20 in HCC using GEO datasets. GSEA analysis revealed some tumor pathways associated with RGS20, namely DNA REPAIR, mTORC1, MYC TARGETS V1 signaling being predominant. In addition, the RGS20 gene is correlated with five lincRNAs (LINC00511, PVT1, MIR4435-2HG, BCYRN1, and MAPKAPK5-AS1). Besides BCYRN1, the other four lincRNAs present possible interaction with RGS20 protein and only lincRNA PTV1 showed potential interaction with mRNA of RGS20. This evidence supports that RGS20 was found to be significantly associated with some tumor-related signaling pathways and long non-coding RNAs (lincRNAs) that exhibit oncogenic potential. An interesting study using overexpression and knockdown of RGS20 in different cancer cell lines showed that it may play a role in the regulation of cancer cell migration and invasion, and even perhaps metastasis [19].

Of interest, all of the lincRNAs (LINC00511, PVT1, MIR4435-2HG, BCYRN1, and MAPKAPK5-AS1) that we found associated with RGS20 have previously been implicated in HCC. For instance, a high expression of LINC00511 was found in HCC tissues and cell lines, and blocking the LINC00511 contributed to a lower proliferation, migration, and invasion in HCC cell lines [33]. Similarly, PVT1 has been shown to facilitate the growth of HCC cells via the PVT1/EZH2/miR-214 axis [34]. In the case of MIR4435-2HG, its expression was found to be upregulated in HCC which may promote cancer cell proliferation by upregulating miRNA-487a [35]. The high expression of BCYRN1 was also linked to an unfavorable prognosis in patients with HCC [36]. The expression of MAPKAPK5-AS1 was also significantly increased in HCC, and it was suggested that the MAPKAPK5-AS1/PLAGL2/HIF-1α signaling loop contributes to HCC progression [37]. Since some cancers were not associated with RGS20 and some also did not show a significant difference, despite RGS20 being expressed highly in their respective tissues (e.g., glioblastoma). This can be partially explained by variability in the expression of certain genes in different tissues. Furthermore, a cumulative effect of (epi-) genomics and oncogenic networks/mechanisms might be contributing to this. Recently, a novel immunodiagnostic assay was developed to screen tumor-associated antigens (TAAs) associated with HCC, that includes RGS20 in a panel of eleven TAAs (AAGAB, C17orf75, CDC37L1, DUSP6, EID3, PDIA2, RGS20, PCNA, TAF7L, TBC1D13, and ZIC2) [38]. Thus, providing further evidence to support our study indicating the distinctive involvement of RGS20 in HCC. Overall, our results suggest that RGS20 is an attractive candidate to predict the prognosis for survival of HCC patients. Further studies in experimental and clinical settings are required to validate our findings.

## 5. Conclusions

The regulator of G protein signaling 20 correlates with lincRNAs harboring oncogenic potential and is markedly upregulated in hepatocellular carcinoma. Our analysis further supports the putative function of RGS proteins, particularly RGS20, in cancer.

## Figures and Tables

**Table 1:** Logistic regression assessment of RGS20 expressions between the clinical variable groups using TCGA data.

Clinical Characteristics	Odd Ratio (OR)	*p*-Value
Age (≥65 vs. <65)	0.923 (0.494–1.722)	0.801
Gender (male vs. female)	0.780 (0.398–1.518)	0.465
Grade (G3 + G4 vs. G1 + G2)	2.358 (1.245–4.539)	0.009 **
Stage (III + IV vs. I + II)	0.743 (0.337–1.612)	0.454
AFP (≥400 vs. <400)	2.360 (1.048–5.619)	0.043 *
Child-pugh (B + C vs. A)	0.767 (0.262–2.166)	0.617
Fibrosis (no fibrosis vs. fibrosis)	0.808 (0.412–1.573)	0.531

* *p* < 0.05, ** *p* < 0.01.

**Table 2:** Univariate survival prediction of RGS20 expression and the clinical factors using TCGA data.

	Univariate Cox Regression	
	HR (95% CI of HR)	*p* Value
Age (continuous)	1.029 (1.004–1.054)	0.022 *
Gender	0.761 (0.420–1.379)	0.368
Grade	1.405 (0.912–2.164)	0.123
Stage	1.463 (1.072–1.997)	0.017 *
Child-pugh	1.447 (0.611–3.426)	0.401
AFP (continuous)	1.000 (1.000–1.000)	0.615
Fibrosis	0.686 (0.380–1.239)	0.212
RGS20 (continuous)	77.931 (5.954–1019.956)	<0.001 ***

* *p* < 0.05, *** *p* < 0.001.

## Data Availability

The data set in this study can be found in the https://portal.gdc.cancer.gov/repository (accessed on 30 November 2021) and https://www.ncbi.nlm.nih.gov/geo/ (accessed on 31 December 2021).
